# Delayed presentation of Subclavian venous thrombosis following undisplaced clavicle fracture

**DOI:** 10.1186/1749-7922-3-25

**Published:** 2008-07-22

**Authors:** Tony Kochhar, Chethan Jayadev, Jay Smith, Emmet Griffiths, Kamaljit Seehra

**Affiliations:** 1 Department of Trauma & Orthopaedics, Queen's Hospital Romford, Essex, UK; 2 Department of Trauma & Orthopaedics, Hillingdon Hospital, Middlesex, UK; 3 Department of Trauma and Orthopaedics, Norfolk and Norwich University Hospital, Colney Lane, Norwich, NR4 7UY, UK

## Abstract

Medial clavicle fractures are uncommon, accounting for approximately 5 percent of all clavicle fractures. Vascular injuries are uncommon but are recognised as either an immediate complication due to transection of the vessel by the displaced fracture, or as a late complication, secondary to compression from abundant callus formation. We present an unusual case of positional venous insufficiency in the upper limb as an immediate complication of a closed, minimally displaced clavicle fracture, with secondary subclavian venous thrombosis formation eleven days following the injury.

## Background

Injuries to the clavicle are very common and account for up to 10% of all fractures[2]. Midshaft and lateral third clavicle fractures are common sporting injuries; the vast majority present without neurovascular injury and proceed to uncomplicated union [1-3]. Fractures to the medial clavicle are uncommon, accounting for only 2–5% of all clavicle fractures [2-5] and are often due to high energy injuries. The medial clavicle protects the brachial plexus, subclavian and axillary vessels and the superior lung. Fractures can therefore be complicated by damage to these structures.

Much of the literature and research has concentrated on midshaft and distal clavicle fractures and acromioclavicular joint injuries. We aim to highlight the difference in mechanism of injury and complications associated with medial third clavicle fractures.

## Review of the literature

In general, vascular injuries following clavicle fractures are uncommon but are recognised as either an immediate complication due to transection of the vessel by the displaced fracture [6,7], or as a late complication, secondary to compression from abundant callus formation. There have been several reported cases of venous insufficiency associated with clavicle fractures [8-11] and of acute compression of the subclavian vessels following displaced midshaft fractures[12]. There have also been reported cases of neural injury associated with these common injuries [13].

Isolated injuries of the medial end of the clavicle are uncommon and are usually part of multisystem injuries[14]. Throckmorton and Kuhn presented a review of all clavicle fractures treated at their institution over a five year period. Out of 614 clavicle fractures, only 57 were identified as medial third fractures. 80% of these occurred in middle aged men. Just over 80% of these injuries were associated with motor vehicle accidents (53% were passengers/driver of a vehicle; 16% were pedestrians hit by a vehicle; 11% motorcycle accidents). Ninety percent of cases were defined as having multi-system trauma. Associated injuries included haemothorax or haemopneumothorax (42%), pulmonary contusions, respiratory failure or adult respiratory distress syndrome (55%), rib fractures (73%) with only one patient suffering from a vascular complication of subclavian vein perforation following a gunshot wound. The other complications reported with fractures of the medial clavicle were similar to those seen in association with lateral and midshaft fractures: skin or soft tissue complications, malunion, nonunion and refracture.

In children, physeal fracture of the medial end of the clavicle has been documented with a range of associated complications occurring acutely by the initial injury or chronically following a missed diagnosis [15]. Hoarseness [16], thoracic outlet syndrome[17], pneumothorax[18], tracheo-oesophageal fistula[19], and venous thrombosis have also been reported[7]. Blunt subclavian injury has been documented following fracture of the clavicle usually associated with high energy trauma[20], however one case has been documented of it occurring following an epileptic fit. In this particular instance the patient presented as critical ischaemia of the upper limb [21].

## Discussion

Medial clavicle fractures are uncommon injuries and have therefore been largely overlooked. The available literature points out that medial third clavicle fracture, like scapular body fractures, are commonly associated with a high-energy blunt trauma mechanism of injury and should therefore prompt the treating physician to look for other associated poly-trauma. There is a high association with mortality from multi-system trauma. In such situations, cardio-respiratory injuries and compromise appear to be the most frequent and most serious associated injuries. Other injuries to neck/thoracic viscera have been reported.

While there have been several reports of vascular injury (laceration, compression; acute or late) following fractures of the clavicle, the vast majority have been due to displaced fractures of the midshaft or lateral end. However, most midshaft and lateral clavicle fractures are not associated with injuries to other structures. Such fractures heal completely without complication with non-operative management. This review reiterated the inherently different nature of medial third clavicle compared to the more common midshaft or lateral third fractures. Medial third clavicle fractures are more likely to be associated with poly-trauma and serious complications, which can easily be overlooked, particularly after the immediate post-injury phase. This paper also emphasises the need for repeated vigilance following initial clinical assessment.

## Competing interests

The authors declare that they have no competing interests.

## Authors' contributions

TK conceived the idea and wrote the paper. CJ, JS and EG were instrumental in analysing the notes, collecting the data and inserting images. KS was responsible for editing and approving the final manuscript.

**Figure 1:**
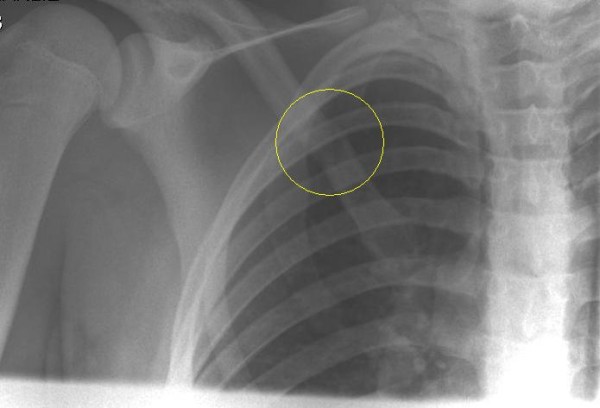
AP X-Ray AP X-Ray showing an undisplaced fracture.

**Figure 2:**
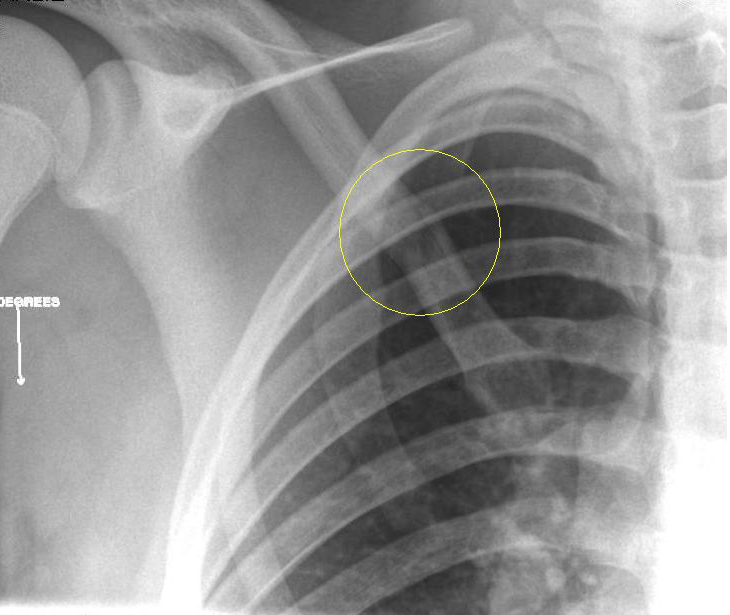
AP X-Ray showing an undisplaced fracture of the medial end of the clavicle.

**Figure 3:**
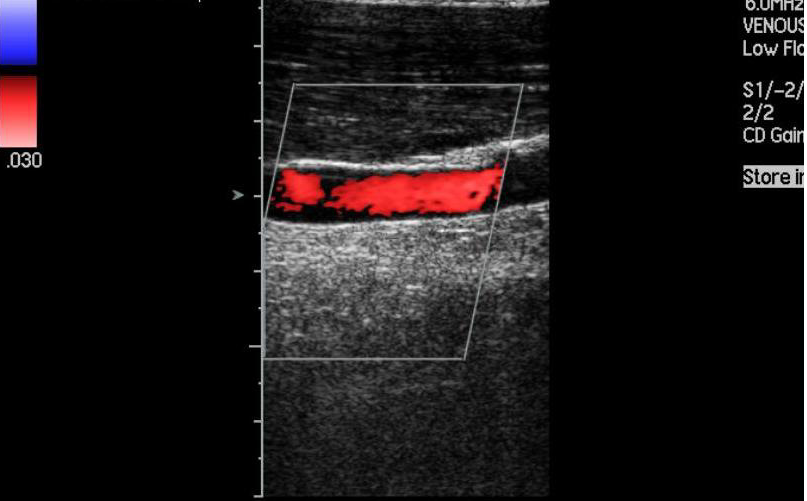
Duplex scan – arm in flexion demonstrating vascular compromise.

**Figure 4:**
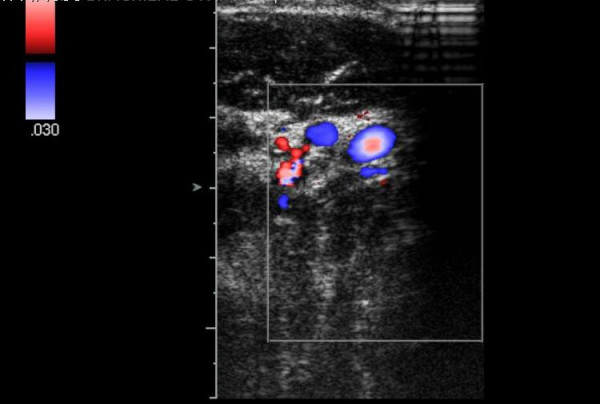
Initial duplex scan – arm straight.

**Figure 5:**
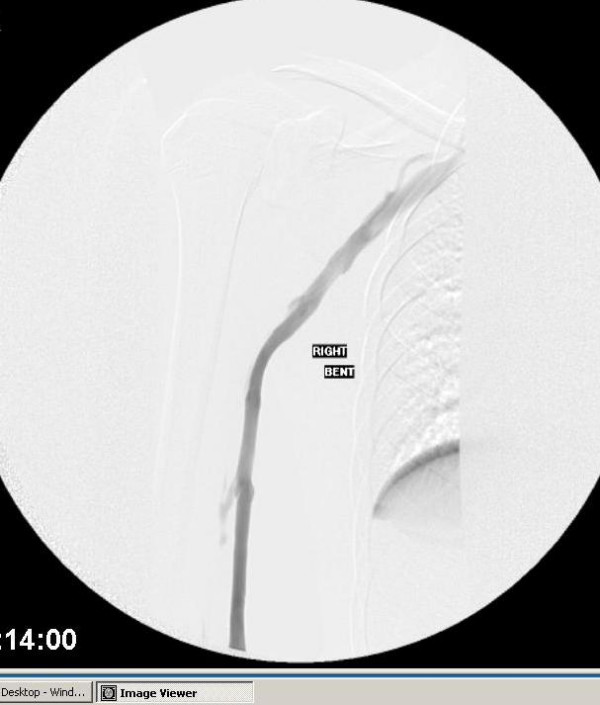
Venogram with arm flexed. This shows normal venous flow without occlusion.

**Figure 6:**
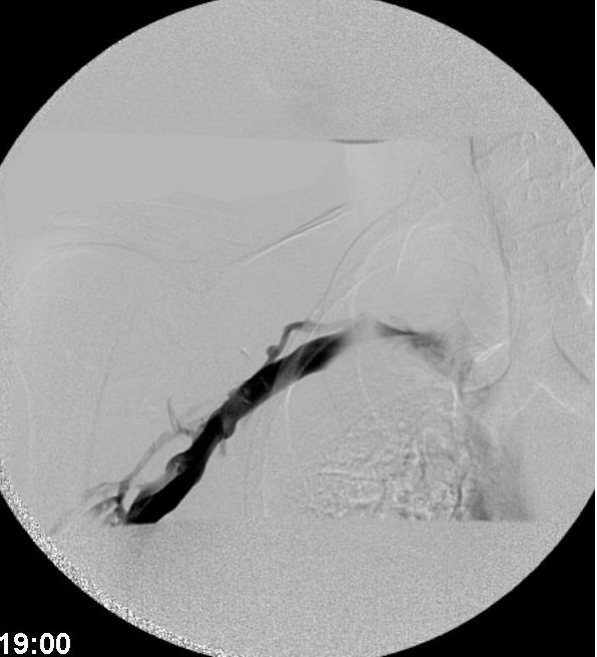
Venogram image demonstrating poor axillary flow.
